# Novel Effector Protein EspY3 of Type III Secretion System from Enterohemorrhagic *Escherichia coli* Is Localized in Actin Pedestals

**DOI:** 10.3390/microorganisms6040112

**Published:** 2018-10-27

**Authors:** Mariano Larzábal, Wanderson Marques Da Silva, Nahuel A. Riviere, Ángel A. Cataldi

**Affiliations:** Instituto de Biotecnología CICVyA, Instituto Nacional de Tecnología Agropecuaria (INTA), Los Reseros y Nicolás Repetto, Hurlingham Buenos Aires 1686, Argentina; marques.wanderson@inta.gob.ar (W.M.D.S.); riviere.nahuel@inta.gob.ar (N.A.R.); cataldi.angeladrian@inta.gob.ar (Á.A.C.)

**Keywords:** pathogenic *Escherichia coli*, type III secretion system, effectors, actin polymerization, pedestals

## Abstract

Enterohemorrhagic *Escherichia coli* (EHEC) and enteropathogenic *Escherichia coli* (EPEC) are attaching and effacing (A/E) pathogens, which translocate effector proteins to intestinal enterocytes through a type III secretion system (T3SS). T3SS and most of its effector proteins are encoded in a pathogenicity island called LEE. Recently, new effectors have been located outside the LEE. This study aimed to characterize EspY3, a novel non-LEE encoded T3SS effector of EHEC. EspY3 shares homology with SopD and PipB2 effector proteins of Salmonella’s T3SS-1 and T3SS-2, respectively. The presence of recombinant EspY3 in the supernatant samples demonstrated that EspY3 was secreted by the T3SS of EHEC and EPEC. Through infection assays, we demonstrated the translocation of EspY3 into Caco-2 cells by T3SS of EPEC. The subcellular localization of EspY3 was determined in the pedestal region, where its presence generates a significant increase in the size of the pedestals area. The EspY3 effector induced the elongation of polymerized actin pedestals in infected Caco-2 by EPEC. This study confirmed that EspY3 is part of the repertoire of T3SS effectors of EHEC O157:H7, and that it participates in modeling cellular actin during the infection.

## 1. Introduction

Enterohemorrhagic *Escherichia coli* (EHEC) and enteropathogenic *Escherichia coli* (EPEC) are an important cause of food and water-borne illnesses in humans worldwide. EHEC infections cause hemorrhagic colitis and can result in the potentially fatal hemolytic uremic syndrome because of the presence of Shiga toxins [1,2,3]. Although EPEC do not possess the Shiga toxins genes, they belong to the attaching and effacing (A/E) bacterial pathogens family, as well as EHEC. The formation of the attaching and effacing lesion involves the intimate attachment of the bacteria to the hosts intestinal epithelial cells, the effacement of intestinal microvilli, and the formation of actin-filled protrusions or ‘‘pedestals’’ [4]. The ability of these pathogens to generate the A/E lesion is given by the type III secretion system (T3SS), which is encoded in the pathogenicity island called locus of enterocyte effacement (LEE) [5]. The T3SS translocates virulence factors from the bacteria to the host cells cytoplasm. These virulence factors are known as effectors and each pathogen has a diverse repertoire of such effectors to enable a specific infection process. One of the most representative effectors of EPEC and EHEC is Tir (translocated intimin receptor), which is encoded inside the LEE. The assembly of pedestals through the actin-cytoskeleton reorganization is initiated with the secretion of Tir by T3SS. Tir phosphorylation allows its incorporation into the epithelial cell membrane, where it interacts with the bacterial outer membrane adhesin (Intimin) [6,7], thereby recruiting the host’s signaling molecules Nck, N-WASP and the Arp2/3 complex, which catalyze actin polymerization [8,9]. LEE-encoded effectors of EHEC and EPEC are sufficient to cause the A/E lesion.

Recently, new effectors with different roles during cell subversion have been described. Many of these effectors are located outside the LEE, and thus, some of them are called Nle (non-LEE encoded). These effectors mimic and replace eukaryotic protein activities subverting cellular functions to allow successful colonization of the host [10,11]. For instance, EspM modulates Rho GTPases [12]; NleH and NleD inhibit apoptosis [13]; NleB, NleC, NleE, and NleH interfere with the inflammatory signaling pathway [14,15,16]; and EspF, EspH and EspJ inhibit phagocytosis [17,18,19]. However, about half of the LEE genes lack homologues and have not been functionally studied.

Tobe et al. [20] identified 60 genes encoding for predicted T3SS effectors secreted by EHEC O157:H7 and confirmed some of these proteins as part of the repertoire of T3SS by protein translocation assays. In addition, an important identified family of EHEC effector proteins composed of five proteins denoted as EspY (from EspY1 to EspY5), contain an N-terminal WEX5F domain that presents homology with SopD (*Salmonella* outer protein D), a well characterized T3SS-1 (SPI-1) effector protein of *Salmonella* [21,22]. SopD has been shown to contribute in gastroenteritis, systemic virulence, and persistence of *S. typhimurium* infection in the studied animal models [23,24]. SopD acts cooperatively with SopB (Inositol phosphate phosphatase) in the “ruffling” and rearrangements of cellular actin, resulting in the manipulation of the membrane host cell during the invasion, allowing the infection of polarized epithelial cells [25]. Other authors have confirmed EspY1 and EspY4 as genuine translocated T3SS effectors of EHEC O157:H7, whilst EspY5 was characterized as a pseudogene [20]. Blasche et al. [25], demonstrated the participation of EspY1 in apoptosis and regulation of cell cycle. Therefore, the aim of study was to demonstrate that the putative effector protein EspY3, predicted as a potential virulence factor of the EspY family, is translocated by the T3SS of EHEC O157:H7 and EPEC O127:H6.

## 2. Materials and Methods

### 2.1. Bacterial Strains and Medium

The strains used in this study were EHEC O157:H7 EDL933 and EPEC O127:H6 E2348/69. EHEC O157:H7 EDL933 was isolated from Michigan ground beef linked to the outbreak of 1982 that proceeded from contaminated hamburgers. On the other hand, EPEC O127:H6 E2348/69 was isolated in Taunton, United Kingdom, in 1969, during an outbreak of infantile diarrhea. Both bacteria have a functional T3SS. EHEC O157:H7 Rafaela II strain (Δ*escN*) obtained in our laboratory was used as a T3SS negative control. All strains were routinely grown in Luria-Bertani (LB) broth or Dulbecco’s modified Eagle’s medium (DMEM) with 4500 mg/L glucose, L-glutamine, and sodium pyruvate, without sodium bicarbonate aerobically at 37 °C. When required, the medium was supplemented, with 50 µg/mL ampicillin.

### 2.2. Primers Design, PCR Amplification and EspY3 Cloning

The *espY3* gene (ECs0472 according to Sakai strain genome annotation) was amplified by PCR from EHEC O157:H7 EDL933 strain as a template. The primers were designed based on the genome sequence of EHEC O157:H7 Sakai strain. The sequences of the primers were as follows: EspY3F: 5′GGATCCATGAAAACCACTCAC′3 and EspY3R: 5′ACTAGTGTCGACGAACTACAT′3. The PCR amplification of *espY3* (1572 bp) was carried out at a final volume of 50 µL containing 5 µL of bacterial lysate (template), MgCl_2_ 1.5 mM, 0.2 mM dNTPs, 1 µM primer and Taq DNA Platinum polymerase 1.5 U. The PCR conditions were: 94 °C 2 min (1 cycle); 94 °C 30 s, 50 °C 30 s, 72 °C 1 min (30 cycles). The coding sequence of *espY3* was cloned into *pGEM-T Easy* vector (ampicillin resistance) in *E. coli* XL-1 strain. The *pGEM*-*espY3* construction was digested with SpeI and BamHI restriction enzymes, and the fragment was subcloned into *pLF-3xFLAG* (ampicillin resistance). *pLF-3xFLAG* vector was provided by Dr Diego Comerci (IIB-INTECH-Institute of Biotechnological Research-UNSAM). The cloned segment was sequenced at the Genomic Unit from the Biotechnology Institute (CICVyA-INTA) to confirm its sequence. EspY3 was recombinantly expressed to the FLAG tag in O157:H7 EDL933, EPEC O127:H6 E2348/69 and EHEC O157:H7 Rafaela II (Δ*escN*).

### 2.3. Detection of Recombinant Secreted Effectors to the Culture Supernatant

The expression and subsequent secretion of the recombinant effector through T3SS was assessed by growing strains EHEC O157:H7 EDL933, EPEC O127:H6 E2348/69, and EHEC O157:H7 Rafaela II (T3SS mutant) transformed with *pLF-3xFLAG-espY3* in DMEM, with 0.5 mM of Isopropyl β-D-1-thiogalactopyranoside (IPTG). After 4 h of incubation under agitation (200 rpm) at 37 °C, the cultures were centrifuged at 10,000 rpm for 10 min at 4 °C. The supernatants were filtered (0.22 µm pore-size) and the proteins were precipitated with trichloroacetic (TCA) or acetone at a final concentration of 10% *v*/*v*, followed by incubation at −20 °C for 16 h. The supernatants were centrifuged at 10,000 rpm for 20 min at 4 °C. Then, the secreted proteins were resuspended in Tris-HCl pH 8.8 and SDS 2× loading buffer was added to the resuspended proteins. The recombinant protein was detected using 1:2000 anti-FLAG mouse specific antibodies and 1:10,000 peroxidase conjugated goat anti-mouse IgG as secondary antibody in *Western blot* assays.

### 2.4. Translocation Assay and Fluorescence Confocal Microscopy

Human epithelial colorectal adenocarcinoma cells (Caco-2) were grown in DMEM supplemented with 10% heat-inactivated fetal bovine serum and 2 mM glutamine, at 37 °C with 5% CO_2_ in a glass support (Nunc^®^ Lab-Tek^®^ II Chamber Slide ™ system, SIGMA-ALDRICH, Boston EEUU) up to 70–90% confluence. For the translocation assay, 5 ×10^4^ cells per well were seeded in 8-well plates and incubated for 24 h at 37 °C with 5% CO_2_. EHEC O157:H7 EDL933 and EPEC O127:H6 E2348/69 transformed with *pLF-3xFLAG-espY3* were grown in LB broth overnight, and then diluted in 400 µL of DMEM with 1 mM of IPTG, at a multiplicity of infection (MOI) of 100 or 5 for EHEC and EPEC, respectively. Caco-2 cells were infected for 4–5 h at 37 °C in 5% CO_2_, and then gently washed three times with PBS to remove non-adherent bacteria. After that, the cells were fixed with 4% paraformaldehyde during 5 min. cellular actin was stained with Rhodamine-phalloidin (Red), the eukaryotic nuclei and bacterial DNA with TO-PRO-3 (Blue), and the recombinant proteins were detected using anti-FLAG mouse specific antibodies and Alexa 488-conjugated goat anti-mouse IgG as secondary antibody (Green). The area of the pedestals was determined by observing 15 randomized fields in triplicate for each sample. The Image J software was used to delineate and calculate the pedestals area generated by both strains. The cells were observed under fluorescence microscopy with a confocal laser microscope (40× objective-Leica TCS SP5- CICVyA-INTA).

## 3. Results

### 3.1. Bioinformatic Analysis of EspY3

**Figure 1:**
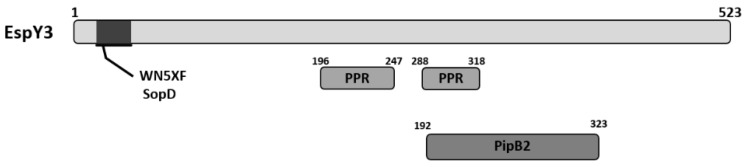
Schematic representation of EspY3 protein domains. EspY3 protein presents a WEX5F domain in the N-terminal region, which group with SopD T3SS-1 effectors family of *Salmonella*. In addition, EspY3 shows homology to PipB2, a T3SS-2 effector protein of *Salmonella*.

Tobe and colleagues observed that EspY3, which is encoded in a pathogenic island outside the LEE, has a N-terminal WEX5F domain, and that this protein groups with SopD, a T3SS-1 effector of *Salmonella* [20]. In addition, through BLASTp search we have observed that EspY3 shows homology with PipB2 effector protein of *Salmonella* that is translocated by T3SS-2 (Figure 1). EspY3 also presents two copies of 8 and 9 tandem of pentapeptide repeats (PPR) that contain the consensus sequence A(N/D)(L/M/F)XX (Figure 1). Although this feature is also present in PipB2, PipB2 has more copies of PPR (30 copies) that are located in the C-terminal region; the function of these repeats remains unknown in this protein [26]. PPR family has over 500 members in the prokaryotic and eukaryotic kingdoms, and the function of these repeats is uncertain in most proteins [27].

### 3.2. Expression and Secretion of Recombinant EspY3 Protein by T3SS

**Figure 2:**
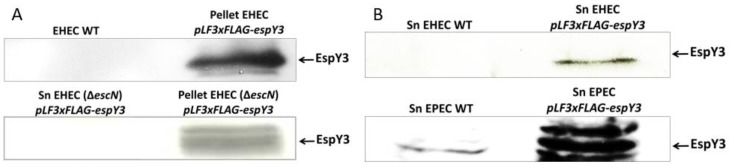
Expression and secretion of recombinant EspY3. (**A**) EspY3 (61 KDa) was cytoplasmically expressed (pellet) by EHEC O157:H7 EDL933 and EHEC O157:H7 Rafaela II (Δ*escN*) in DMEM, with 0.5 mM of IPTG. EspY3 was undetectable in EHEC O157:H7 Rafaela II (T3SS mutant) supernatant. (**B**) EspY3 was observed in the precipitated DMEM medium (supernatant) of EHEC and EPEC 0.5 mM of IPTG. Sn: supernatant of EHEC or EPEC. WT: EHEC or EPEC wild type. Anti-FLAG mouse IgG and anti-mouse peroxidase conjugated (Goat) were used as primary and secondary antibodies, respectively, for western blot assays.

Transformed EHEC strains expressed the recombinant cytoplasmic EspY3, as evidenced by western blot assays (Figure 2A). Then, precipitated supernatant samples of EHEC and EPEC bacteria were used to confirm the secretion of EspY3 protein through the membrane by T3SS (Figure 2B). The presence of EspY3 in the supernatant showed that this protein was secreted by the T3SS of both EHEC and EPEC. In contrast, recombinant EspY3 was absent from EHEC O157:H7 Rafaela II (T3SS mutant) supernatant. Thus, the protein needs to be recognized and secreted by the T3SS (Figure 2A).

### 3.3. Subcellular Localization of EspY3 by Confocal Microscopy

**Figure 3:**
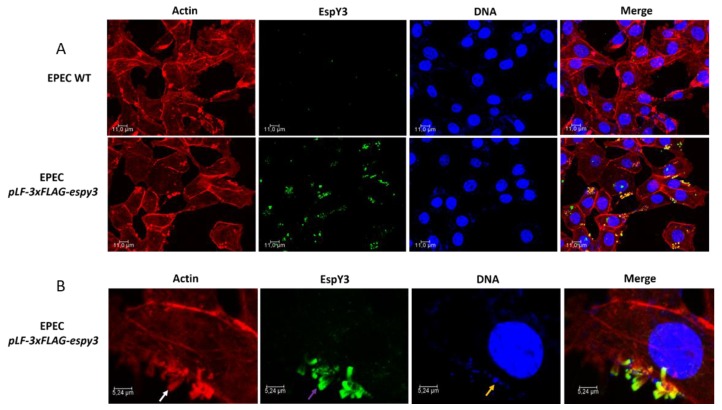
Subcellular localization of EspY3. (**A**) Caco-2 cells were infected with EPEC wildtype (WT) and EPEC pLF-3xFLAG-espy3 (MOI 5). The polymerized cellular actin was stained with Rhodamine-phalloidin (Red), the eukaryotic nuclei and bacterial DNA with TO-PRO-3 (Blue), and EspY3 protein was detected using anti-FLAG mouse specific antibodies and Alexa 488-conjugated goat anti-mouse IgG as secondary antibody (Green) (ZOOM 1). (**B**) The white arrow shows the polymerized cellular actin (pedestals), the purple arrow shows the translocated EspY3, and the blue arrow shows the presence of EPEC on the pedestal lesion. The yellow color in the last image (merge between the red color of the pedestals and the green color of the EspY3) represents the co-localization of EspY3 with the polymerized actin in the pedestals (ZOOM 3). The cells were observed under a confocal laser scanning microscopy (40×, 1.5 AN objective-Leica TCSSP5-CICVyA-INTA).

To determine if T3SS of EHEC translocates EspY3 to the cytoplasm, we infected Caco-2 cells monolayers with EHEC O157:H7 EDL933 *pLF-3xFLAG-espy3* (MOI of 100) in DMEM medium supplemented with IPTG to allow the induction of the recombinant protein expression. After 4 h of incubation at 37 °C in 5% CO_2_, the recombinant protein was undetectable using specific anti-FLAG epitope-antibodies (Data not shown). We also performed the same assays for EPEC *pLF-3xFLAG-espy3* (MOI of 5). In this case, microcolonies of polymerized actin pedestals were evident for EPEC WT and EPEC *pLF-3xFLAG-espy3*; which demonstrates the ability of these bacteria to produce lesions on the epithelial cells (Figure 3A). EspY3 translocation occurred in the epithelial cells (green color) (Figure 3A), and this protein had been co-localizated with the polymerized cellular actin (yellow color) (Figure 3B). Therefore, the subcellular location of EspY3 is associated to the pedestal region.

### 3.4. Elongation of Polymerized Actin Pedestals by EspY3

**Figure 4:**
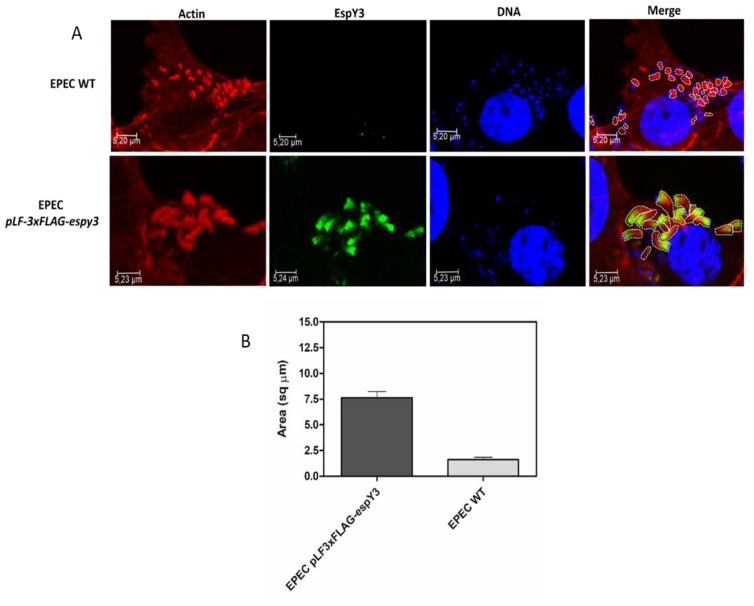
Calculation of the pedestals areas. (**A**) The Image J software was used to delineate and calculate the pedestals area generated by EPEC WT and EPEC *pLF-3xFLAG-espy3* on infected Caco-2 cells. The polymerized cellular actin was stained with Rhodamine-phalloidin (Red), the eukaryotic nuclei and bacterial DNA with TO-PRO-3 (Blue), and EspY3 protein was detected using anti-FLAG mouse specific antibodies and Alexa 488 conjugated goat anti-mouse IgG as secondary antibody (Green) (ZOOM 3). (**B**) The graph represents the significant difference (*p* < 0.05) in the increase of the pedestals formed by the translocation of EspY3.

*Image J* software was used to delineate and calculate the pedestals area generated by both EPEC WT and EPEC *pLF-3xFLAG*-*espy3* (Figure 4A)*.* This comparison showed that the pedestals produced by EPEC *pLF-3xFLAG*-*espy3* by the translocation of EspY3 have a significantly increased area compared to the EPEC WT pedestals (Figure 4B).

## 4. Discussion

We have demonstrated that EspY3 is secreted into the environment in a T3SS-dependent manner in EHEC O157:H7. Even more, EspY3 was recognized and secreted by the T3SS of EPEC O127:H6, although the EspY effector family is not part of the EPEC genomic repertoire. Many reports indicate that substrates of T3SS are recognized by the similar mechanism of another pathogen, for example, YopE effector protein of *Yersinia* can be secreted by T3SS of *Salmonella* [28]. Similarly, IpaB T3SS effector protein of *Shigella* can be secreted by the T3SS of *Salmonella* [28], and the heterologous ExoS secretion of *Pseudomonas aeruginosa* by *Yersinia pseudotuberculosis* has also been reported [29]. In addition, the T3SS from *Erwinia chrysanthemi* expressed in *E. coli* was able to export AvrB and AvrPto substrates from *Pseudomonas syringae* [30]. It has also been shown that IncA, IncB, and IncC. of *S. flexneri* were secreted by a type III mechanism *in Chlamydiae* [31]. The mechanism of T3SS heterologous substrates recognition has not yet been resolved, but some researchers have proposed the presence of signal regions in the first 15 codons of yop mRNA effectors of *Yersinia*, and suggested that the secretion could be mediated by a chaperone-mediated export pathway [32,33]. EPEC and EHEC are closely related as they have a homologous T3SS, and they share a large number of LEE-encoded effectors, such as Tir, Map, EspB, EspF, EspH, EspZ, and EspG. Therefore, it could be expected that a T3SS EHEC effector family as EspY would be recognized as an effector by the T3SS of EPEC.

Our studies revealed that EspY3 was translocated by the T3SS in epithelial cells infected with EPEC *pLF-3xFLAG*-*espy3*. EspY3 was localized in the pedestal region, where its presence was responsible for a significant increase in the pedestals area. Although EspY3 subcellular location has been described here, its possible effector function in the pedestal region remains to be determined. However, a higher amount of polymerized actin below the site of contact of the bacteria with the cell would imply strong adherence. The bacteria that have the EspY3 effector could have an advantage during colonization of the host. In this way, a more intimate adhesion would allow the bacteria to persist for longer in the intestine. The presence of the SopD domain may be considered in the possible subversion of cellular actin, since it participates in the “ruffling” during the cellular invasion of *Salmonella* [34]. Regarding its function in the cell, we believe that the SopD domain of EspY3 has a direct relation with several host signaling molecules responsible for participating in the recruitment of actin, such as Nck, N-WASP, and the Arp2/3 complex during the formation of the A/E lesion. Mutagenesis assays directed to the SopD domain should be carried out to determine its participation in the modeling of cellular actin. Furthermore, the participation of PipB2 and PPR motifs in the modeling of cell actin dynamics could not be ruled out.

No EspY3 translocation to epithelial cells was observed by EHEC. Probably, the low number of lesions caused by EHEC on the epithelial cells compared to EPEC makes it difficult to observe the translocation and subcellular localization of the recombinant protein. In addition, a lower expression of EspY3 was observed in culture supernatants of EHEC compared to EPEC. This could also influence the impossibility to detect the translocated EspY3 in EHEC. EPEC has an adherence factor (EAF) coded in a plasmid called bundle-forming pilus (BFP). BFP is implicated in the formation of bacterial autoaggregates, and in the localized adherence to cultured epithelial cells [35]. Thus, a greater amount of infecting microcolonies of bacteria and a higher protein expression would allow the translocation of a large amount of recombinant EspY3.

Altogether, in this work, we have confirmed that EspY3 is an effector protein translocated by the T3SS of both EHEC O157:H7 and EPEC O127:H6. We have also demonstrated that EspY3 localizes in the pedestal region in EPEC. The efforts to identify potential virulence factors through genomic and proteomic studies will allow a better understanding of the bacterial pathogenesis of EHEC. Subsequently, understanding the effectors function on cellular components provides further insight into the mechanisms that cause disease, and therefore, increase our knowledge on cell biology.
